# Neuroimaging of Lightning-Related Cerebral Demyelination: A Case Report

**DOI:** 10.7759/cureus.106668

**Published:** 2026-04-08

**Authors:** Manoj Kumar Nayak, Rahman Ataur, Biswamohan Mishra, Pradosh Kumar Sarangi, Suprava Naik, Biswajit Sahoo

**Affiliations:** 1 Radiodiagnosis, All India Institute of Medical Sciences, Bhubaneswar, IND; 2 Neurology, Kalinga Institute of Medical Sciences, Bhubaneswar, IND; 3 Radiodiagnosis, All India Institute of Medical Sciences, Deoghar, IND

**Keywords:** brainstem demyelination, cerebral white matter, demyelination, lightening strike, steroid therapy

## Abstract

High-voltage electrical injuries from lightning are not only responsible for causing fatal burns but can also cause various neurological abnormalities. Herein, we present a case of a middle-aged woman who was struck by lightning and presented with disorientation and persistent vomiting. The magnetic resonance imaging (MRI) of the brain revealed confluent, symmetrical T2/fluid-attenuated inversion recovery (FLAIR) hyperintensities in the white matter of both cerebral hemispheres and the brainstem. She received steroids for brain inflammation, and her symptoms improved. This is a rare case of lightning-induced diffuse cerebral and brain stem demyelination.

## Introduction

Lightning strikes, delivering voltages from thousands to millions of volts, are often fatal, with approximately 30,000 lightning-related deaths reported in India over the past decade [1]. The extent of injury depends on voltage, exposure duration, and tissue resistance. Nerves, muscles, and blood, rich in water and electrolytes, are low-resistance conductors, making the central nervous system (CNS) particularly vulnerable. While neurological complications such as cerebral hemorrhage, infarction, and seizures are documented, diffuse cerebral and brainstem demyelination is exceedingly rare. Previous literature reports delayed or localized demyelination following high-voltage lightning strikes, but diffuse involvement is unreported [2,3]. This case describes a rare presentation of lightning-induced diffuse CNS demyelination.

## Case presentation

A middle-aged woman was struck by lightning while outdoors, sustaining full-thickness burns over her neck and anterior chest. She was disoriented immediately after the incident and developed persistent, small-volume, watery vomiting. There was no loss of consciousness, seizures, or other concomitant injuries. Her medical history was unremarkable. On examination, she was conscious but oriented only to person, not to time or place. Vital signs were stable: afebrile, pulse of 98/min, blood pressure of 136/78 mmHg, and SpO2 of 99% on room air. Dermatological examination revealed 10-15% full-thickness burns over the anterior chest, sternal region, medial borders of both breasts, and lateral neck, consistent with lightning injury patterns. During the neurobehavioral examination, the patient was fully conscious, alert, and cooperative. She was oriented to a person but not to time or place. Attention was adequate, and she was able to follow simple verbal commands without difficulty. Speech was fluent and coherent, with no dysarthria or language deficits. Comprehension was intact, and there were no hallucinations, delusions, or perceptual disturbances. Affect was appropriate, and mood appeared stable. Immediate recall was preserved, although there was mild impairment in recent memory. Remote memory was intact. Higher cortical functions, including praxis and visuospatial abilities, were grossly preserved. Her motor power was 5/5 in all four limbs (Medical Research Council scale), muscle tone was normal in all limbs, and cranial nerves I-XII were intact. Deep tendon reflexes were 2+ and symmetrical, with no pathological reflexes. The primary neurological symptoms were disorientation and persistent vomiting.

**Figure 1:**
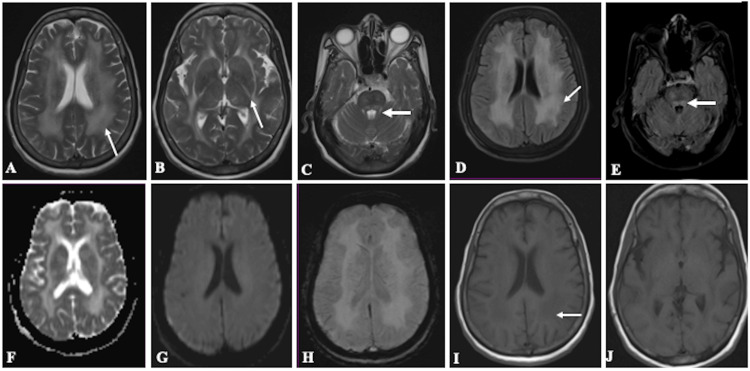
MRI of the brain images of the patient with lightning strike MRI: magnetic resonance imaging; T2W: T2-weighted; FLAIR: fluid-attenuated inversion recovery; ADC: apparent diffusion coefficient; SWI: susceptibility image Axial T2W (A-C) and FLAIR (D, E) images showed confluent symmetrical hyperintensities in subcortical, deep, and periventricular white matter of bilateral frontoparietal lobes, bilateral external and internal capsules (thin white arrows in A, B, D), and pons (thick white arrows in C, E). Axial ADC (F) and diffusion-weighted images (G) showed no restricted diffusion. Axial SWI (H) showed no evidence of blooming. Axial T1 images (I, J) showed that white matter lesions were mildly hypointense (thin white arrow in I)

Due to persistent disorientation and vomiting, a brain magnetic resonance imaging (MRI) was performed. The MRI showed confluent, symmetrical T2/fluid-attenuated inversion recovery (FLAIR) hyperintensities in the subcortical, deep, and periventricular white matter of bilateral fronto-parieto-temporal lobes, bilateral internal and external capsules, medulla, and pons, without diffusion restriction (Figures 1A-1J). No evidence of hemorrhage, infarction, or mass effect was noted. These findings were suggestive of demyelination, likely induced by the lightning strike. The symmetrical white matter hyperintensities raised considerations of toxic-metabolic encephalopathy, viral encephalitis, or acute disseminated encephalomyelitis (ADEM). However, the absence of fever, prodromal illness, or cerebrospinal fluid analysis (not performed due to rapid clinical improvement) argued against encephalitis. The temporal association with a lightning strike and lack of exposure to toxins made demyelination secondary to electrical injury the most likely diagnosis. The patient’s burns were managed with surgical debridement, suturing, and regular dressings. She received intravenous fluids and antibiotics to prevent infection. For neurological symptoms, she was treated with dexamethasone (4 mg twice daily) to reduce potential neuroinflammation. Within five days, her disorientation and vomiting resolved. The steroid dose was tapered, and she was discharged on day 7 without neurological deficits. At discharge, the patient was neurologically intact. Outpatient follow-up at one month confirmed sustained resolution of symptoms, with no recurrence of vomiting or disorientation. Repeat MRI was not performed due to clinical recovery, but neurological monitoring was recommended to assess for delayed complications.

## Discussion

Lightning strikes cause approximately 6,000-24,000 deaths annually worldwide, with India being a hotspot due to its monsoon-driven thunderstorm activity [1]. CNS injuries are underreported due to high mortality and limited access to advanced imaging in rural areas. Lightning delivers immense electrical and thermal energy, causing injury through direct neuronal damage, disruption of the blood-brain barrier, glutamate excitotoxicity, oxidative stress, and ischemic damage from vascular spasms [2,3].

The MRI showed confluent, symmetrical T2/FLAIR hyperintensities without diffusion restriction, consistent with demyelination, a rare lightning sequelae. Unlike prior delayed/patchy cases, this diffuse cerebral and brainstem involvement is novel [2]. Normal motor examination despite extensive MRI changes indicates predominant cognitive/autonomic manifestations (disorientation, vomiting) over focal deficits, likely from myelin disruption or neuroinflammation. This aligns with Chen et al.’s immediate prolonged electrical injury category [4].

Dexamethasone mitigated presumed neuroinflammation, yielding rapid recovery. Though no trials exist for lightning-induced demyelination, corticosteroids are first-line in analogous conditions like ADEM due to anti-inflammatory effects [5-7]. We used a moderate dose (4 mg twice daily for five days with taper), extrapolated from demyelinating syndromes, monitoring for hyperglycemia, infection, and gastrointestinal/neuropsychiatric effects [8,9]. Therapy should be tailored to the individual patient with careful follow-up. The absence of diffusion restriction on MRI suggests reversible pathology, consistent with the patient’s outcome. Clinicians should consider early MRI in lightning strike survivors with neurological symptoms to guide management and prognosis. In the present case, a repeat MRI was not obtained because the patient demonstrated complete clinical recovery, with resolution of disorientation and vomiting by day 5, and remained neurologically intact at one-month follow-up. Under such circumstances, further neuroimaging was unlikely to influence management and was therefore deferred to avoid unnecessary cost and exposure, in line with a patient-centered and resource-conscious approach. Nevertheless, the patient was advised long-term neurological monitoring, with follow-up imaging reserved for any recurrence or emergence of new neurological symptoms. This is particularly important given the risk of delayed CNS complications, including epilepsy and motor disorders [10].

Acute toxic leukoencephalopathies may present as confluent, widespread, bilateral, and symmetric hyperintensities in the white matter observed on T2-weighted and FLAIR MRI sequences, impacting both supratentorial and infratentorial regions. These findings are predominantly associated with inhaled heroin consumption, methadone toxicity, or the administration of high-dose intrathecal methotrexate [11]. The patient in our case had no history of such exposure. Hypertensive encephalopathy usually presents as posterior reversible encephalopathy syndrome (PRES), primarily impacting the parieto-occipital lobes [12]. Considering that the patient lacks a history of hypertension and the imaging findings were not predominantly posterior, PRES was considered unlikely. Instead, the current case exhibited widespread involvement of the cerebrum and brainstem. Leukoencephalopathy with brain stem and spinal cord involvement constitutes an adult-onset leukodystrophy that may manifest as gradually progressive cerebellar ataxia and spasticity, accompanied by dorsal column dysfunction. Imaging generally demonstrates diffuse, bilateral periventricular white matter hyperintensities on T2-weighted sequences. Patients with a mild variant of vanishing white matter disease may present with clinical features including migraines, psychiatric disturbances, cerebellar dysfunction, spasticity, and seizures. Neuroimaging typically reveals diffuse, bilateral white matter hyperintensities and evidence of cystic degeneration [13]. Since the onset of symptoms was acute in this case, adult leukodystrophies, which are characterized by a chronic and indolent course, are considered less probable.

## Conclusions

Rare neurologic complications like diffuse cerebral and brainstem demyelination can occur due to lightning strikes. In those affected individuals, early MRI is essential for the evaluation of CNS abnormalities. Steroid therapy has the potential to aid in recovery; however, further research is required. Long-term follow-up is advised for these patients to monitor for delayed complications.
